# Impact of Antibiotic Therapies on Resistance Genes Dynamic and Composition of the Animal Gut Microbiota

**DOI:** 10.3390/ani11113280

**Published:** 2021-11-16

**Authors:** Tony Rochegüe, Marisa Haenni, Stanislas Mondot, Chloé Astruc, Géraldine Cazeau, Tristan Ferry, Jean-Yves Madec, Agnese Lupo

**Affiliations:** 1 Unité Antibiorésistance et Virulence Bactériennes (AVB), ANSES—Université de Lyon 1, 31 Avenue Tony Garnier, 69007 Lyon, France; tony.rochegue@hotmail.fr (T.R.); marisa.haenni@anses.fr (M.H.); Jean-Yves.MADEC@anses.fr (J.-Y.M.); 2 Micalis Institute, AgroParisTech, Université Paris-Saclay, INRAE, 78350 Jouy-en-Josas, France; stanislas.mondot@inrae.fr; 3 3C-G Clinical, 69238 Saint-Symphorien-sur-Coise, France; astruc.chloe@gmail.com; 4 Unité Epidémiologie et Appui à la Surveillance (EAS), ANSES—Université de Lyon 1, 69007 Lyon, France; geraldine.cazeau@anses.fr; 5 Service des Maladies Infectieuses et Tropicales, CHU de Lyon, Hôpital de la Croix-Rousse, 69004 Lyon, France; tristan.ferry@chu-lyon.fr; 6 Centre International de Recherche en Infectiologie, CIRI, Inserm U1111, CNRS UMR5308, ENS de Lyon, UCBL1, 69007 Lyon, France

**Keywords:** bovines, pigs, poultry, horses, dogs, cats, intestinal microbiota, beta-lactams, macrolides, bacitracin, fluoroquinolones, tetracycline, companion animals, food-producing animals

## Abstract

**Simple Summary:**

Antibiotics perturb the gastrointestinal microbiota by killing bacteria beneficial for animal health and favoring the emergence of potential pathogens. Furthermore, antibiotics favor the emergence of resistant bacteria. Current knowledge on animals’ intestinal microbiota and effects of antibiotics is blurred by the various posology, administration routes, and implemented methodologies for its analysis. We summarized 71 studies analyzing the administration of antibiotics by different routes, conducted on the main food-producing and companion animals, highlighting differences in the methodology applied for the intestinal microbiota and antibiotic resistance analysis. Overall, therapeutic dosage decreased bacterial species diversity and richness in the microbiota and selected antibiotic resistance genes. For non-therapeutic dosage, information on the selection of antibiotic resistance was scarce and the effect on the intestinal microbiota scattered. Understanding the gut microbiota composition and function in animals could open up strategies for its modulation to improve animal health and performance, and to minimize the negative impact of antibiotics.

**Abstract:**

Antibiotics are major disruptors of the gastrointestinal microbiota, depleting bacterial species beneficial for the host health and favoring the emergence of potential pathogens. Furthermore, the intestine is a reactor of antibiotic resistance emergence, and the presence of antibiotics exacerbates the selection of resistant bacteria that can disseminate in the environment and propagate to further hosts. We reviewed studies analyzing the effect of antibiotics on the intestinal microbiota and antibiotic resistance conducted on animals, focusing on the main food-producing and companion animals. Irrespective of antibiotic classes and animal hosts, therapeutic dosage decreased species diversity and richness favoring the bloom of potential enteropathogens and the selection of antibiotic resistance. These negative effects of antibiotic therapies seem ineluctable but often were mitigated when an antibiotic was administered by parenteral route. Sub-therapeutic dosages caused the augmentation of taxa involved in sugar metabolism, suggesting a link with weight gain. This result should not be interpreted positively, considering that parallel information on antibiotic resistance selection was rarely reported and selection of antibiotic resistance is known to occur also at low antibiotic concentration. However, studies on the effect of antibiotics as growth promoters put the basis for understanding the gut microbiota composition and function in this situation. This knowledge could inspire alternative strategies to antibiotics, such as probiotics, for improving animal performance. This review encompasses the analysis of the main animal hosts and all antibiotic classes, and highlights the future challenges and gaps of knowledge that should be filled. Further studies are necessary for elucidating pharmacodynamics in animals in order to improve therapy duration, antibiotic dosages, and administration routes for mitigating negative effects of antibiotic therapies. Furthermore, this review highlights that studies on aminoglycosides are almost inexistent, and they should be increased, considering that aminoglycosides are the first most commonly used antibiotic family in companion animals. Harmonization of experimental procedures is necessary in this research field. In fact, current studies are based on different experimental set-up varying for antibiotic dosage, regimen, administration, and downstream microbiota analysis. In the future, shotgun metagenomics coupled with long-reads sequencing should become a standard experimental approach enabling to gather comprehensive knowledge on GIM in terms of composition and taxonomic functions, and of *ARG*s. Decorticating GIM in animals will unveil revolutionary strategies for medication and improvement of animals’ health status, with positive consequences on global health.

## 1. Introduction

Awareness of the effect of antibiotic therapies on the composition of the gastrointestinal microbiota (GIM) and selection of antimicrobial resistance (AMR) is continuously rising. Antibiotics are considered as major disruptors of the GIM, depleting butyrate-producing species beneficial for the host health and favoring the emergence of potential pathogens, resulting in the so called “dysbiosis”. This negative effect of antibiotic therapies is observed in humans [1,2,3] and in animals, as well [4,5,6,7]. The presence of antibiotics in the intestine exacerbates selection of resistant bacteria that can disseminate in the environment and propagate to further hosts [8,9,10,11,12]. This issue is of particular importance in food-producing animals, which can excrete resistant bacteria in the environment, which in turn can further propagate to other hosts through the environment or the food chain [13,14].

All antibiotic classes clinically implementable nowadays do not exert the same selective pressure on the GIM and AMR, and the same molecule has different effects according to the administration route [15,16]. In animals, antibiotics are commonly administered by oral route. According to the fifth report of the World Organization for the Animal Health (OIE), tetracyclines were the most widely used antibiotics in terrestrial food-producing animals, followed by macrolides and penicillins. In companion animals, aminoglycosides were the most frequently used antibiotics, followed by penicillins and cephalosporins, in 2017 [17]. Data reported from 31 European countries by the ESVAC (European Surveillance of Veterinary Antibiotic Consumption) focused on food-producing animals in 2018, and indicated tetracycline as the most sold antibiotic (in mg/PCU), followed by penicillins and sulfonamides [18]. These antibiotics are used to treat different infections including gastro-intestinal, respiratory, and skin infections. Despite all the measures engaged to regulate antibiotic consumption in food-producing animals to counteract AMR development, 26% of countries (n = 42/160) participating in the survey conducted by the OIE, used antibiotics as growth promoters. Most of these countries were located in Americas, Africa, Asia, Far East, and Oceania. Bacitracin, flavomycin, and avilamycin were the first three antibiotics most commonly used with this specialty. In countries where antibiotics are used as growth promoters, regulation is often lacking. However, also in these countries, actions to discourage usage of antibiotics as growth promoters are in progress [17]. Furthermore, studies to understand the impact of growth promoters on GIM and antibiotic resistance genes (*ARG*s) selection are increasing.

Most GIM studies focus on the large intestine, using stools, which are considered representative of the GIM and constitute non-invasive sampling [19,20]. Before the 1990s, GIMs studies were mostly based on cultivation of bacterial isolates [21].

Improvement of both molecular biology techniques and sequencing platforms have opened new possibilities to explore the GIM, allowing deeper access to the diversity of the microbial population. Gene-targeted and shotgun sequencing are currently gold standard approaches in microbiota research [22]. The first molecular approaches consisted in sequencing one of the hypervariable regions (V1–V9) of the 16S rRNA operon (rDNA) [23,24,25]. Nowadays, microbiome studies that relate to 16S rDNA gene approaches are mostly sequencing the near full length of the operon, combining short and long-reads sequencing [26,27]. In general, obtained and assembled sequences are assigned to the so-called “Operational Taxonomic Units” (OTUs); each of these will include sequences with 97% nucleotide identity with each other and, in turn, will show similarity to known 16S rDNA sequences. More recently, amplicon sequencing variants (ASV) or ribosomal sequencing variants (RSV) were implemented for microbiota analysis [28].

Parameters often used to describe GIM are richness, consisting in the absolute number of OTUs in each sample and diversity. Diversity intrinsic to the sample is referred to as alpha-diversity, often estimated by the Shannon index, whereas diversity between samples is addressed as beta-diversity, often estimated by the Bray–Curtis index [29].

The first shotgun metagenomic analysis was realized in 2006 [30]. This approach provides information on the overall composition of GIM, with the possibility to predict functions assumed by the diverse bacterial taxa, reliably. Shotgun still requires more complex bioinformatics and computational efforts than the 16S rDNA approach.

Studies on the GIM are often associated with quantitative PCR (qPCR) approaches that are designed to detect and quantify genes of interest. This approach achieves higher specificity and sensitivity compared to 16S rDNA and shotgun sequencing. It is relatively inexpensive and does not require sophisticated bioinformatics skills [31].

Irrespective of the molecular biology approach, important biases in GIM studies can derive from sampling and conservation. Bad storage of stools (temperature, +/− buffer) can induce DNA degradation and thus reduce the quality of downstream analysis. Other important biases could be introduced during DNA extraction from feces. PCR inhibitors in DNA extracted from animals’ stools are particularly difficult to remove. For all these reasons, the International Human Microbiome consortium has provided standards for samples collection, storage, and DNA extraction procedures [32,33,34,35,36,37]. These standardizations should be adapted also for GIM studies in animals to improve harmonization of methodologies and allow comparison among studies.

Finally, some studies analyzing the effects of antibiotics on GIM have exploited animal models [38] and also bioreactors mimicking the GIM [39]. This approach allows a controlled quantification of antibiotics to which bacteria are exposed but often relies on a simplification of the bacterial community, thus impairing ecological conclusions of antibiotics action on GIM.

Current knowledge on animals GIM and antibiotic interactions is blurred by diversity of posology, administration routes, and implemented methodologies for its analysis. Besides, the pharmacodynamics of each antibiotic varies according to the animal host [40], rending the picture more complex compared to humans [41].

In this review, we analyzed studies conducted on animals, focusing on the main food-producing and companion animals. The main findings reported on the action of antibiotics on *ARG*s selection, but also on the GIM composition of animals, were summarized. The analysis encompasses diverse classes of antibiotics, highlighting differences according to the molecule, the administration route, and the methodology applied for the AMR and GIM analysis. The search for original research papers in the PubMed library, accessed during July–August 2021, was conducted using the keywords “antibiotics [animal host] microbiota”. Animal host consisted of calves, bovines, pigs, poultry, ovine, rabbits, horses, dogs, and cats. No studies were found for ovine and rabbits. Seventy-one papers were considered as containing relevant data, as relative studies were conducted analyzing the GIM and *ARG*s before and after the antibiotic therapy. Studies including the analysis of antibiotic effects on one species or genus, or an *ARG*, were included, as well.

The summary of the studies highlights: (i) if animals were hosted in experimental or commercial farms for food-producing animals; (ii) the administration route and posology of the antibiotics; (iii) the methodology applied for analyzing the GIM composition and *ARG*s; and (iv) main observed effects.

In the following paragraphs, the content is organized according to the animal host and, when pertinent, for each host according to the antibiotic class.

## 2. Calves

The main components of calves’ GIM are *Ruminococcaceae* and *Lachnospiraceae* (40%) (Firmicutes phylum), and *Bacteroidaceae* (15%) (Bacteroides phylum), followed by Enterobacterales (25%) (Proteobacteria phylum), which decreases during GIM maturation (5%), whereas *Prevotellaceae* increases (20%) (Bacteroidetes phylum). The composition of feedcolostrum and GIM in neonate calves is similar, and GIM’s evolution occurs rapidly during the first 10 weeks of life [42,43,44].

Amounts of *ARG*s were found higher in calves than in adult animals reared in the same environment. Living conditions, such as wet soil and the number of cattle residing in the farm (>500), were risk factors for colonization with cefotaxime (third generation cephalosporin, GC) resistant bacteria [45]. A decrease of Enterobacterales during the first weeks of life has been associated with a general decrease in *ARG*s abundance in calves, with breed influencing the abundance of certain *ARG*s and *ampC* gene (copy number) [45].

### 2.1. Effect of Waste Milk Feeding on Calves’ GIM

Exposure of calves GIM to antibiotics is frequent through feeding waste milk containing antibiotics residual. Penati et al. [46] observed that calves fed with milk containing residual cefalexin (first GC) differed for GIM composition, with a final higher abundance of Chlamydiae phylum compared to an untreated group until 6 weeks after cefalexin residual fed withdrawal. Dupouy et al. [47] investigated the selective power of cefquinome (fourth GC) administered to calves colonized by different amounts of Extended-Spectrum-Beta-Lactamase (ESBL) producing *E*. *coli*. The administration mimicked residual concentration found in waste milk (2 mg/L) and that of udder milk of treated cows (20 mg/L). Cefquinome had a selective effect, irrespective of the administrated concentration, on all calves colonized by ESBL-producing *E*. *coli* prior to antibiotic exposure. Maynou et al. [48] compared the GIM of Holstein calves receiving raw milk and a formula with waste milk containing residues of beta-lactams and probably lincosamides at unknown concentrations. No difference between the two groups was observed in terms of GIM composition. Effects of low-concentration antibiotics (penicillin, ampicillin, and oxytetracycline) were associated with a decrease of certain microbial functions, such as stress response, regulation of the cell signaling, and nitrogen metabolism, in neonatal GIM of treated calves, potentially affecting the adaptation of GIM to environmental challenges [49]. In another study, a very low concentration of a cocktail of antibiotics in waste milk did not alter calves’ GIM composition at the phylum level. Besides, a significant decrease of the *Veillonella* genus was observed in calves exposed to antibiotic residues compared to an unexposed control group [50].

### 2.2. Therapeutic Concentration of Antibiotics in Calves

#### 2.2.1. Beta-Lactams

In 6-months-old Norwegian Red calves treated by intramuscular injection (IMI) of benzyl-penicillin, Grønvold et al. [51] observed the emergence in *E*. *coli* of resistance to benzyl-penicillin and to other classes of antibiotics, whereas no resistance was observed in non-treated calves. Considering that *E*. *coli* has low permeability to benzyl-penicillin, the mechanisms underlining the emergence of such resistance remain unclear.

Antibiotic therapy based on trimethoprim-sulfamethoxazole, a folic acid synthesis inhibitor, followed by ceftiofur (3rd GC), delayed diversification in species composition of calves GIM, whereas inter-individual variability, which usually decreases with maturation, remained overall elevated, suggesting that antibiotics delayed maturation of the GIM [52].

#### 2.2.2. Original Data on the Analysis of Amoxicillin Effects on Calves’ GIM

**Figure 1:**
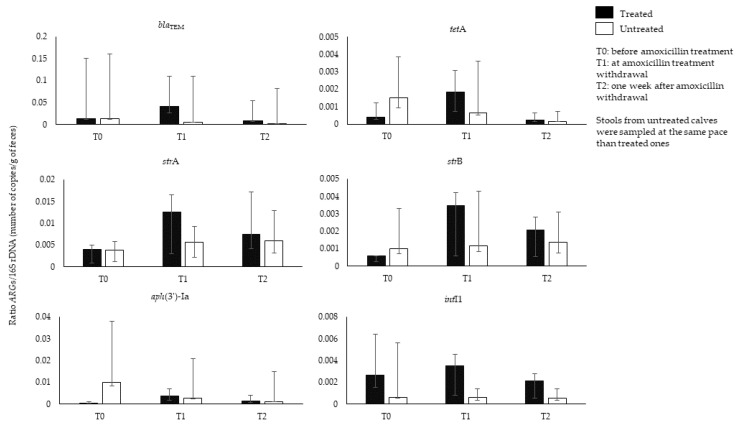
Representation of *ARG*s/16S rDNA ratio (number of copies/g of feces) evaluated by qPCR in feces of treated and untreated calves at amoxicillin pre-treatment (T0), at amoxicillin withdrawal (T1), and 1 week after amoxicillin withdrawal (T2).

History of amoxicillin therapy has been associated to the rise of resistance in *E*. *coli* isolates from calves’ feces [53]. To our knowledge, longitudinal studies analyzing the effects of amoxicillin on calves’ GIM were lacking. We thus prospectively collected feces from calves (n = 16) aged from 5 to 26 days, belonging to five breeds (Charolais/Montbeliard, Montbeliard, Prim’ Holstein, Charolais/Prim’ Holstein, Limousin/Montbeliard), and resident in different farms (n = 7) in the Rhône-Alpes region (France), during the period October 2018–March 2020. Eleven out of 16 calves were suffering from omphalitis (umbilical cord infection) and were treated with IMI of amoxicillin (Longamox^®^, 15 mg/kg) for a duration varying between 4 and 16 days. The remaining five calves did not receive antibiotic treatment, and their feces were sampled at the same pace of the treated ones. The abundance of 41 *ARG*s, *int*I1/2/3, and of 16S rDNA was analyzed by qPCR [54]. Seventeen out of 41 investigated *ARG*s were found in the feces of all calves before amoxicillin treatment. The *bla*_TEM_ gene and bacterial abundance were comparable between the treated and untreated group before treatment (ratio *bla*_TEM_/16S rDNA: 0.013 in both groups). At the end of amoxicillin treatment (T1), the amount of *bla*_TEM_ increased in treated calves (*bla*_TEM_/16S rDNA ratio: 0.040) along with other *ARG*s (*tet*A, *str*A and *str*B), and *int*I1, index of class 1 integrons. These data suggest co-selection by amoxicillin treatment of *ARG*s related to other antibiotic classes and potential multidrug development (Figure 1). A decrease of all *ARG*s was observed 1 week after amoxicillin withdrawal (T2) (*bla*_TEM_/16S rDNA ratio: 0.008). The amount of *bla*_TEM_ constantly decreased in the untreated group (*bla*_TEM_/16S rDNA ratio: T1, 0.005; T2: 0.002). The difference observed in the amount of *ARG*s at pretreatment and post-treatment, or between treated and untreated group, was not statistically significant (Wilcoxon Mann Whitney or Wilcoxon signed-rank test, *p* > 0.05). Several factors could confound the effect of the amoxicillin treatment on the *bla*_TEM_ amount, for instance variation of the *bla*_TEM_ gene amount among individuals, probably due to variation of calves’ age, which ranged from 5 to 26 days. Age is a determinant for GIM composition and *ARG*s amount at early life. In addition, calves were distributed in seven commercial farms probably contributing to the difference in *ARG*s content as well, because of different farm management. Environmental exposure of all calves to *ARG*s cannot be excluded, as calves of the untreated and treated group lived in the same farm, thus probably influencing the level of difference of *bla*_TEM_ amount between the two groups. For a better understanding of antibiotics action on the GIM and selection of *ARG*s, experiments in environmentally controlled set-up would be a benefit for avoiding confounding factors influencing GIM composition and *ARG*s variation further than antibiotic action. However, studies in commercial farms are necessary to model antibiotic therapies effects in a real-life environment and evaluate *ARG*s propagation to other hosts or in the farm environment.

#### 2.2.3. Macrolides

Prophylactic subcutaneous injection (SCI) of tulathromycin caused a decrease of *Bifidobacterium* genus (Actinobacteria) in treated calves [55]. On the contrary, comparison of metaphylactic therapy based on enrofloxacin, a fluoroquinolone, or tulathromycin in Holstein calves did not evidence major changes in the GIM neither at the phylum level nor for gene function. Besides, Desulfovibrionales (Proteobacteria), which include species of potential pathogens for humans [56], had a higher relative abundance in the enrofloxacin-treated group 56 days post-withdrawal [57].

Metaphylactic SCI of tildipirosin did not alter the GIM of Holstein calves, at least at the phylum level [58]. Several antibiotic therapies caused a decrease of GIM diversity and *E. coli* amount during the treatment and until 15 days after withdrawal in Holstein calves observed in three different commercial farms [59].

#### 2.2.4. Tetracyclines

Keijser et al. [60] analyzed the effects of a low and high dose of oxytetracycline in treated calves. The high-dose was administered for 5 days, whereas the low-dose was administered for 7 weeks. Major changes compared to a group of untreated animals were observed over time. Both oxytetracycline doses correlated with a decrease of *Ruminococcus*, *Coprobacillus*, and *Lachnospiraceae*, all belonging to the Firmicutes phylum, along with an increase of *Prevotella* (phylum Bacteroidetes), *Faecalibacterium,* and *Blautia* (phylum Firmicutes), compared to an untreated group. The selection of *tet*M gene and other *ARG*s, such as *mel* and *flo*R, occurred only in high-dose treated calves and lasted for all the study period (42 days). Oultram et al. [61] analyzed the effects of oxytetracycline (IMI), tulathromycin (SCI), and florfenicol (SCI) used to treat pneumonia and otitis occurring in 7-week-old Holstein calves, hosted in a commercial farm. Considering the five most abundant detected genera, *Lactobacillus*, *Faecalibacterium*, *Bacteroides*, *Parabacteroides*, and *Sharpea*, a statistically significant decrease in the oxytetracycline-treated calves was observed for *Lactobacillus* genus compared to the control group. Overall, antibiotic treatment slightly decreased species richness in the calves’ microbiota 1 week after withdrawal. However, no statistical significance was observed compared with control group. Thames et al. [13] studied by qPCR the effects of neomycin and tetracycline orally administered on the abundance of selected *ARG*s (*tet*C/G/O/W/X, *erm*B/F, *sul*1/2; *int*I1), and found that only *tet*O was significantly more abundant in the treated group.

#### 2.2.5. Other Antibiotics

Lhermie et al. [62] analyzed the effect of fluoroquinolones at low (2 mg/L) and high (10 mg/L) doses administered by IMI in young bulls (7–10-months-old) and calves (2–5-weeks-old). The therapy moderately selected for resistant Enterobacterales compared to the untreated group, and with less detectable effects in young bulls, probably because of a more mature GIM compared to calves that was expected to contain less Enterobacterales and more species difficult to cultivate (the study was conducted by cultivation). However, calves were colonized by fluoroquinolone-resistant bacteria before the treatment. Dobrzanska et al. [6] analyzed the effect of thiamphenicol. At 7 days from thiamphenicol administration, Proteobacteria increased because of *E*. *coli* expansion, along with the emergence of *mcr*-2, a less prevalent gene than *mcr*-1 responsible for colistin resistance, and *oqx*B gene, encoding for antibiotic efflux pump. In the treated group, a rise of methanogenic Archaea and *Prevotellaceae*, typically associated to weight gain, was also observed.

## 3. Adult Bovines

Studies conducted on adult bovines are scarce. Recently, Wang et al. [63] provided information on the GIM composition and *ARG*s in yak, beef, and dairy cattle. Composition of the GIM was similar among animals. However, abundance of *ARG*s differed among hosts, with higher abundance in beef and dairy cattle than in yak.

Holman et al. [64] analyzed the effects of a single IMI dose of oxytetracycline and tulathromycin in Angus-Herford cattle in an experimental farm and moved to a feedlot for the study. The moving to the feedlot caused more remarkable changes in the GIM composition than antibiotic therapies. Besides, both antibiotics caused the decrease of several species compared to the control group, and recovery was observed 12 days post-treatment. The *tet*M and *tet*W genes augmented in treated animals and remained higher than in the untreated control group up to 34 days post-treatment.

In general, studies conducted in calves were more numerous than those conducted on adult bovines, probably because antibiotic therapies are more frequent in young animals that suffer more often than adults from diseases such as pneumonia and diarrhea [45,65] (Table 1).

## 4. Pigs

A reference catalogue of pigs’ GIM is available, and the basal resistome of pigs never exposed to antibiotics and residing in experimental farms was provided [66,67,68]. Tetracycline resistance genes are the most abundant in pigs’ resistome, whereas the main components of the microbiota, at the phyla level, are Firmicutes (65.5%), Bacteroidetes (14%), Proteobacteria (10%), and Actinobacteria (7.1%). Piglets have been used as a model for studying neonatal entero-colitic diarrhea [69], probably justifying the large amount of available studies analyzing the GIM.

### 4.1. Beta-Lactams

Effects of antibiotics on pigs’ GIM have been investigated since the early 2000s, exploiting DGGE (Denaturing Gradient Gel Electrophoresis). Besides the limitations inherent to this technique [70], studies converged for effects of IMI-administered amoxicillin, indicating a decrease of Firmicutes and of species diversity and abundance, along with an increase of Proteobacteria in GIM of treated piglets [71,72]. Fouhse et al. [73] investigated the therapeutic effects of amoxicillin administration in piglets with a specific focus on immune system development. A transient increase of Proteobacteria and a decrease of Firmicutes, along with decreased alpha-diversity, were observed in treated animals compared to the untreated group. These differences disappeared 3 days after the withdrawal of the therapy. However, in this study, the administration route of amoxicillin and the region of 16S rDNA sequenced were not explicit. Massacci et al. [74] investigated the effect of amoxicillin in weaned piglets suffering from *E*. *coli* intestinal infection and compared the composition of the microbiota between piglets treated either orally or by parenteral injection, and an untreated control group. Oral administration of amoxicillin produced a dramatic decrease of *Lactobacillus* spp., *Prevotella copri,* and *Ruminococcus*, which are crucial genera for the fiber metabolism, compared to parenteral administration and control groups. After amoxicillin withdrawal, *Lactobacillus* spp. remained more abundant in the control group compared to the two treated groups. Bibbal et al. [75] demonstrated by qPCR that *bla*_TEM_, responsible for ampicillin resistance, increased in feces of pigs after ampicillin administration compared to an untreated group. Furthermore, oral administration significantly increased *bla*_TEM_ excretion compared to the IMI administration. Connelly et al. [76] compared by shotgun sequencing the effect of oral administration of amoxicillin versus intra-venous injection (IVI) of ertapenem. The two antibiotics altered the GIM in different ways: both antibiotics reduced the relative amount of *Faecalibacterium*, a main butyrate-producing genus, *Megasphaera*, *Oxalobacter*, a genus contributing to good health status in humans, but amoxicillin also affected *Lactobacillus* spp.; amoxicillin increased the amount of *Escherichia*, *Bacteroides*, *Fusobacterium*, *Shigella*, and *Klebsiella* genera, suggesting the emergence of potential gastro-intestinal pathogens. Ertapenem increased the relative abundance of *Bacteroides*, *Pseudomonas*, *Enterococcus*, and *Acinetobacter* genera, to which potential gastro-intestinal and extra-intestinal pathogens belong to, as well. Both antibiotics selected *ARG*s. Surprisingly, the results were not compared to those of a non-treated group of piglets. Using a control, untreated groups should not be neglected in the experimental set-up especially when analyzing GIM and *ARG*s of young animals in which GIM is not mature and evolve rapidly. Kouadio et al. [77] studied the effect of amoxicillin administered to piglets orally and by IMI (dose not indicated in the study). By cultivation on selective and non-selective media, the ratio between amoxicillin-resistant and susceptible Enterobacterales was not statistically different when comparing the two administration routes but significantly different between treated and non-treated animals, highlighting the selection of resistant bacteria by amoxicillin whatever the administration route. Lin et al. [78] demonstrated that sub-therapeutic doses of ceftiofur and enrofloxacin caused a higher selection than therapeutic doses of *E*. *coli* resistant to these antibiotics in feces of challenged piglets, irrespective of administration route (IMI or oral). Yun et al. [79] did not find selection of amoxicillin-resistant *E. coli* in piglets treated by amoxicillin (IMI). However, in the latter study, pigs were hosted in commercial farms where the amoxicillin effect could have been confounded by other variables.

### 4.2. Macrolides

IMI of tulathromycin in neonatal piglets did not induce significant differences in diversity of GIM or *ARGs* abundance compared to the untreated control group [80]. On the contrary, a decrease in the diversity of pigs’ GIM composition was observed after lincomycin in-feed administration, favoring the relative raise of Firmicutes and Actinobacteria and decrease of Bacteroidetes and Spirochetes. In particular, a decrease of genera involved in fibers metabolism (*Triponema*, *Succinivibrio*, *Fibrobacter,* and *Cellulosilyticum*) was observed, in favor of an increase of potentially pathogenic genera (*Clostridium*, *Aerococcus*, *Escherichia*, and *Corynebacterium*) in lincomycin-treated pigs [81].

### 4.3. Tetracyclines

Oral administration of oxytetracycline induced an increase of Bacteroidetes and Proteobacteria by expansion of *Prevotella* and *Escherichia* genera, whereas a decrease of Firmicutes occurred along with diversity and species richness. *ARG*s were found increased during treatment, with a decrease sometimes observed after treatment withdrawal [82]. In-feed chlortetracycline in weaned piglets induced a lower species diversity of GIM composition, with an increase of *Lactobacillus* and *Pseudoalteromonas* along with a decrease of *Prevotella*, *Sphaerochaeta*, and *Shuttleworthia* genera. An increase in the abundance of the tetracyclines resistance genes *tet*C/G/Q/W, along with *sul*1/2 and *int*I1/2, was observed in chlortetracycline feed piglets compared to untreated controls [83]. Therapeutic effects of oxytetracycline were studied on piglets to compare IMI and oral administration [16]. In this study, Ricker et al. observed a decrease of Fibrobacteres and Proteobacteria together with an increase of Euryarchaeota and Actinobacteria in GIM of orally-treated piglets, while mild effects were observed in IM-injected piglets. Furthermore, in the oral-administered group, enrichment of genes conferring tetracycline resistance (*tet*W) was observed, and of *aph*2′-id aminoglycosides resistance gene, suggesting a co-selection process. Holman et al. [84] reported a decrease of diversity and richness of the piglets GIM receiving chlortetracycline orally. Zhang et al. [85], comparing the effects of chlortetracycline with those of *Lactobacillus* administration and a non-treated group, showed that Verrucomicrobia that are involved in human gut health homeostasis were less abundant in the chlortetracycline-treated group.

### 4.4. Other Antibiotics

Using IMI as a unique administration route, Zeineldin et al. [86] analyzed the effects of a single dose of different antibiotics, including penicillin, ceftiofur (free acid or conjugated with hydrochloride acid), oxytetracycline, and tulathromycin. All antibiotics caused a shift in diversity and richness of the GIM with tulathromycin and ceftiofur free acid, causing a significant decrease of the relative abundance of the Bacteroidetes phylum. The resilience to a pre-treatment composition was not completely achieved after 14 days for any of the treatments, but especially for ceftiofur free acid and oxytetracycline. The study did not include a non-treated control group.

Oral treatment with colistin of piglets induced a decrease of potential entero-pathogens such as *E*. *coli* or *Shigella* spp., limited to the study period. No mention on AMR effect of colistin administration was reported [87]. Fleury et al. [88] compared the effect of oral administration of colistin in weaned piglets at low and high doses. The major evidence consisted in a decrease of the *E*. *coli* population in the group treated with a high dose, an effect that disappeared at withdrawal. No selection of resistance was observed. This is of relevance for the global therapeutic arsenal, considering that colistin is used as a last-line resort antibiotic in humans and the emergence of plasmid-located resistance mechanisms [89] has put in discussion the usage of this drug in animals. However, long-course colistin therapies could produce different results on *ARG*s selection.

Pissetti et al. reported an increase of Firmicutes together with a decrease of Bacteroidetes, from piglets treated with high antibiotic dosage in cocktail [90]. In this study, piglets not receiving antibiotics were hosted in a separated experimental farm, compared to study groups, which were hosted in commercial farms. Development of multidrug resistance in cultivable *E*. *coli* and enterococci positively correlated with the amount of antibiotic administered to piglets. In-feed flavomycin combined with enramycin was associated to a lower relative abundance of Proteobacteria and Fibrobacteres compared to antibiotic-free pigs [91]. In-feed tylosin was associated to an increase of the Firmicutes/Bacteroidetes ratio and a decrease of Tenericutes compared to control piglets [92] (Table 2).

## 5. Poultry

The predominant phyla occurring in poultry GIM are Firmicutes and Bacteroidetes, followed by Proteobacteria and Actinobacteria, with a proportion of Firmicutes and Bacteroidetes increasing relatively to Proteobacteria with age [93,94]. The easier accessibility to the gastro-intestinal organs by dissection has permitted to better define species components of the different compartments compared to other animals. Diversity of GIM composition increases from the crop to the colon, at least in adult hens [95]. Relevant differences in the GIM composition can be related to breeding management. Indeed, in free-range chicken, Bacteroidetes are the most abundant taxa, whereas conventional-range chicken Firmicutes, and *ARG*s typically found in Firmicutes, are relatively more abundant [94]. In poultry, more antibiotic classes have been analyzed compared to bovines and pigs, including fluoroquinolones.

### 5.1. Beta-Lactams

Penicillin administered as feed additive to 1-day-old chickens resulted in a higher relative abundance of Firmicutes compared to Bacteroidetes at 18 days. Penicillin-fed chicks had higher body weight compared to chickens not receiving the antibiotic [96]. Ampicillin interfered differently with GIM for composition and *ARG*s selection according to administration routes in Leghorn chickens. Oral administration had a higher impact on GIM modification, causing augmentation of Proteobacteria at the expense of Firmicutes phylum. This shift was attributable to an increase of Enterobacterales and in this order by *Klebsiella* and *Escherichia* genera. Higher increase of *ARG*s was observed with oral treatment compared to IMI, although no statistical results were reported for the two routes and relatively to the control group [97].

### 5.2. Streptogramins

Chen et al. [98] compared the effect of in-feed virginiamycin and plant extracts oil on the caecum of young Cobb chickens. Chickens fed with virginiamycin presented lower species diversity compared to oil-fed and control groups, with an increased relative abundance of the Bacteroidetes phylum along with a decrease of Firmicutes. Changes in the metabolome of the caecum were noticed compared to the control group. Dumonceaux et al. [99] previously analyzed the effect of in-feed virginiamycin in the caecum of 50-days-old Cobb chickens. The effect on the diversity of species was less relevant than in the distal part of the intestine compared to the proximal one. In combination with monensin, an anticoccidial, virginiamycin caused a significant increase of the *Escherichia* and a decrease of the *Roseburia* genera relatively to the untreated control in Ross chickens [100].

### 5.3. Tetracyclines

In Lohmann Brown hens hosted in an experimental farm, oral treatment by single or repeated doses of tetracycline and streptomycin caused a decrease in GIM species diversity at 48 h hours post-treatment for both regimens. The sequences representative of enterococci and *E*. *coli* raised, suggesting potential dysbiosis. Restoration of GIM composition to pre-treatment was observed soon after withdrawal. Samples from tetracycline-treated and streptomycin-treated hens were pooled for sequencing analysis, an advantageous strategy for optimizing experimental costs and probably simplifying bioinformatics analysis. However, results obtained for tetracycline or streptomycin-treated chickens were not compared to an untreated control group [93].

In-feed chlortetracycline on the GIM of 42-days-old Arbor Acre chickens caused an augmentation of species diversity in the Firmicutes and Actinobacteria phyla, whereas that of Proteobacteria decreased. An increase of the *Lactobacillus* genus, typically considered as benefic to host health, was observed in the chlortetracycline-treated group [101]. The effect of chlortetracycline, in combination with virginiamycin and amoxicillin, was investigated by Banerjee et al. [102], using sub-therapeutical doses of antibiotics. Compared to a control group fed without antibiotics, Firmicutes, and notably lactobacilli, were relatively more abundant than Bacteroidetes in the antibiotic-fed group. This composition correlated with an increased weight gain in antibiotic-fed chickens.

### 5.4. Fluoroquinolones

Effects of different doses of enrofloxacin on GIM and resistance to *Salmonella* colonization were investigated by Ma et al. [103]. Enrofloxacin-treated chickens demonstrated higher colonization and invasion by *S*. Typhimurium. Furthermore, chickens treated with a high dose had lower abundance of genera beneficial to host health including *Anaerotruncus*, *Butyricicoccus*, and *Ruminococcus* compared to the untreated group. Li et al. [104] analyzed the effects of repeated cycles of enrofloxacin administrations on *S*. Typhimurium challenged chickens. High enrofloxacin dosage eradicated *S*. Typhimurium shedding and caused significant GIM changes compared to low-dosage treatments and untreated groups, with a major increase of *Lactococcus*, *Bacillus* and of Proteobacteria (*Burkholderia*, *Pseudomonas*, *Rhizobium*, and *Acinetobacter* genera). Enrofloxacin at high and low dosage caused the decrease of *Anaerotruncus* genus along with *Blautia*, *Janibacter*, *Flavisolibacter*, and *Parasuterella*, which did not return to the baseline at 7 days post withdrawal. The effects of enrofloxacin on protection against *S*. Typhimurium colonization and invasion were contrasting according to the studies from Ma and Li [103,104]. However, methods to recover *S*. Typhimurium in challenged hosts were different, as well as antibiotic regimen (Li et al. administered repeated antibiotic doses). However, the two studies converged in reporting dramatic changes in the GIM composition of treated animals. Another study found the effect of enrofloxacin consisting in a decrease of microbiota richness, to be transient when administered to 2-week-old Ross chickens, similarly to amoxicillin [105]. In 1-month-old Jing Hong GIM chickens, Elokil et al. [106] studied the effects of enrofloxacin combined with diclazuril, an anticoccidial drug. Overall, Firmicutes, Actinobacteria, Thermi, and Verrucomicrobia phyla decreased in treated chickens, and return to baseline was not observed even at 15 days after withdrawal of treatment.

### 5.5. Bacitracin

Johnson et al. [95] compared the effect of bacitracin administered at sub-therapeutic and therapeutic doses to turkeys. A decrease of species diversity in the caecum was observed with both dosages, although a more dramatic decrease was obtained with therapeutic dose. This difference disappeared over time (42 days). Alteration of the GIM composition was associated to metabolome alteration in treated turkeys, inducing changes potentially beneficial to turkeys’ health. Previously, Díaz Carrasco et al. [107] compared the effects of sub-therapeutic bacitracin to tannins in the caecum of Cobb chickens, sampled at different ages (12, 26, and 30 days old). Differences in species richness were age-dependent. However, in bacitracin-fed chickens, lower species richness was observed at 30 days compared to control and tannins-fed groups, where Firmicutes abundance relatively to Bacteroidetes was higher. Proctor and Phillips [108] analyzed the effects of bacitracin at therapeutic dosage in the colon and caecum microbiota of 30-days-old Cornish/Rock chickens. Both treated and untreated control groups demonstrated a GIM composition mainly constituted of Firmicutes, followed by Proteobacteria, Actinobacteria, and Bacteroidetes. This latter phylum, differently from other studies, occurred with a relative abundance < 1%. Effects of bacitracin concerned mostly the colon microbiota. In the caecum, at the class level, Clostridia increased, whereas *Peptostreptococcaceae* decreased.

### 5.6. Other Antibiotics

Avilamycin caused decreased diversity in the ileum of treated chickens compared to the control group, while no difference to control was observed at the caecum level [109].

Prophylactic administration of several antibiotics to Ross chickens resulted in modification of the ileum and caecum GIM. In particular, an increase of *Enterococcaceae* was observed in chickens treated with amoxicillin or thiamphenicol [110] (Table 3).

## 6. Horses

Horses are considered both food-producing and companion animals, thus they are potential reservoir of AMR for humans by direct contact. In Australia, high occurrence of tetracycline resistance genes was reported [111], whereas in Europe prevalence of third and fourth GC resistance is high in *E*. *coli* from healthy horses’ feces, with medication recognized as risk factors for its occurrence [112]. De Lagarde et al. evidenced also that residing in a riding school and being in contact with >5 caring persons were potential risk factors for ESBL-producing *E*. *coli* colonization of horses.

Benzyl-penicillin effect by IMI was evaluated on hospitalized horses. Development of resistance in *E*. *coli* could not be firmly attributed to antibiotic therapy, considering that resistance also developed in *E*.*coli* from non-treated horses. Differences in GIM composition after benzyl-penicillin treatment were not reported, while an increase of Bacteroidetes, *Clostridioides perfringens,* and enterococci was observed after hospitalization [113]. Costa et al. [7] investigated the effects of penicillin, ceftiofur, and trimethoprim-sulfadiazine on the GIM of mares. Trimethoprim-sulfadiazine caused the most relevant reduction of Verrucomicrobia amount. However, since trimethoprim-sulfadiazine was the only drug administered orally, a real comparison among the different molecules was hindered. At treatment withdrawal (25 days), Proteobacteria amount decreased along with an increase of Firmicutes, suggesting resilience of the GIM. Harlow et al. [114] enumerated colonies of cellulolytic bacteria, lactobacilli, *Salmonella,* and *Clostridioides difficile,* when GIM of horses was challenged by IVI of ceftiofur or oral trimethoprim-sulfadiazine. Both antibiotic administrations significantly decreased the amount of cellulolytic bacteria and lactobacilli, whereas an augmentation of *Salmonella* and *C. difficile* was observed, compared to untreated horses. More recently, Álvarez–Narváez et al. [115] demonstrated the effect of oral erythromycin together with rifampin administered to horses suffering from subclinical pneumonia. Compared to a control group, a decrease of *Rhodococcus equi* and a general increase of *ARG*s copy numbers, suggesting an in-GIM selection of existing *ARG*s, were observed in treated horses. Similarly, Arnold et al. [116] reported a decrease of cecal and fecal microbiome diversity of five horses receiving metronidazole directly in the caecum; however, the results were not compared to those of an untreated control group. Another study showed that horses with diarrhea induced by an antibiotic treatment had an altered microbiota composition compared to horses not receiving antibiotics or those that did not develop post-antibiotic treatment diarrhea [117]. In horses, cellulolytic bacteria residing in the GIM seemed to be most affected by antibiotic treatments, undergoing a decrease in abundance. This decrease could negatively affect horses’ health, considering that these bacteria are crucial to digest fibers, which are the main components of horses’ diet.

## 7. Dogs and Cats

A catalog of the dogs’ GIM is available and has unveiled a large similarity to that of humans with Firmicutes, Bacteroidetes, Proteobacteria, Actinobacteria, and Fusobacteria as main phyla [118].

Pilla et al. [119] analyzed the effects of metronidazole, a broad spectrum antibiotic and anti-parasitic used to treat diarrhea, in the GIM of 1–10-years-old healthy dogs of different breeds, and in parallel the effect of changing diet. Metronidazole caused a decrease of Bacteroidetes and Fusobacteria and an increase of Proteobacteria and Actinobacteria. Abundance of Firmicutes remained constant but composition changed by diminishing Clostridiales and increasing Lactobacillales. At 42 days after antibiotic withdrawal, GIM composition returned to the baseline, except for the Fusobacteria abundance. This phylum in dogs is associated to good health status. Similar results for Fusobacteria and metronidazole administration were found by Igarashi et al. [120], who also reported an increase of Actinobacteria. When metronidazole combined with spiramycin was administered to diarrheic dogs, no difference was found in the amount of certain genera in the GIM compared to dogs administered with a nutraceutical compound [121].

Another commonly administered antibiotic to cure dogs’ diarrhea is tylosin. Manchester et al. [122] observed a decrease of *Fusobacteriaceae* and *Veillonellaceae* and *Bacteroidaceae*, together with an increase of *Enterococcaceae*, after tylosin treatment. At tylosin withdrawal, return to the baseline was individual-dependent. Similarly, Suchodolsky et al. [123] reported long-lasting modification of dogs’ GIM by tylosin treatment with a decrease of Fusobacteria, Bacteroidales, and *Moraxella*, parallel to an increase of enterococci, *Pasteurella* spp., and *Dietzia* spp.

Grønvold et al. [124] analyzed the effects of amoxicillin on dogs’ GIM, which was enriched with Enterobacterales, with *E*. *coli* isolates exhibiting higher rate of resistance compared to isolates recovered at the pre-treatment sampling. A similar selection of resistant *E*. *coli* isolates was found by Werner et al. [125] in diarrheic dogs treated with amoxicillin-clavulanic acid. Amoxicillin and the amoxicillin-clavulanic acid combination affected in a similar way the species diversity and richness of treated dogs. Besides, amoxicillin-clavulanic acid reduced gut commensal taxa, such as *Roseburia*, *Oscillospira, Dialister,* and *Lachnospiraceae* along with increase of *E. coli*. This drugs combination selected ampicillin-resistant *E. coli* and enterococci [126].

Longitudinal studies reporting the effects of antibiotics on cats’ GIM are lacking. To the best of our knowledge, one report described the effect of clindamycin and showed, in clindamycin-treated cats, a decrease of Actinobacteria, Bacteroidetes, *Ruminococcaceae*, *Veillonellaceae,* and *Erysipelotrichaceae* along with an increase of *Clostridiaceae* and Proteobacteria [127] (Table 4).

## 8. Conclusions

Overall, in all animal hosts and irrespective of antibiotic classes, a decrease of species diversity and richness was reported after treatment. In most cases, GIM composition demonstrated resilience, returning to baseline condition for composition and *ARG*s amount after treatments withdrawal. The time necessary to the baseline return was variable among studies.

At sub-therapeutic doses, certain genera belonging to the Firmicutes phylum and involved in sugar metabolism augmented in animals receiving antibiotics in food, suggesting a link with weight gain. This finding cannot be considered as an encouraging result, because usage of antibiotics even at low concentration is linked to the augmentation of *ARG*s in the intestine by selection and by trigging genetic transfer events with negative consequences for global health. The enhanced food-producing animals’ performance obtained using antibiotics as growth promoters could be achieved by replacing antibiotics with modulation of the GIM with probiotics, for instance. For this reason, it is necessary to increase studies in the field, in order to unveil not only GIM composition but also metabolic processes assumed by taxa enriched during antibiotic administration.

At therapeutic doses, bloom of genera hosting potential pathogens was reported recurrently. The risk of this negative effect of antibiotic therapies is ineluctable. Besides, duration of the therapy and dosage could play a role in modulating the intensity of this side effect. In parallel to dysbiosis, selection of *ARG*s occurs, as well. Generally, selective action and consequences on GIM composition were lower for parenteral administration, but effects could still be detected. Indeed, those drugs with hepatic metabolism can reach the gut not-metabolized, together with their metabolites, through bile secretion [128]. Such effects, besides being drug-dependent, are also dependent on pharmacodynamics parameters proper to each animal species [129]. More studies are necessary to clarify these aspects in order to improve therapy duration, antibiotic dosages, and administration routes in the effort of mitigating negative effects of antibiotic therapies. In addition, this review highlights that studies on aminoglycosides are almost inexistent; this is a serious knowledge gap that should be filled considering that aminoglycosides represent the first most commonly used antibiotic class in companion animals worldwide.

Based on the current knowledge, it is difficult to choose an antibiotic, or an antibiotic class, that could have less negative effects compared to others and in the meantime serve as successful treatment. Comparison among studies is hindered by variations in the experimental design including drug concentration, antibiotic combination, therapeutic regimen, and duration of the treatment. Harmonization of experimental procedures is crucial, as well. In fact, studies analyzing animal GIM are often conducted by sequencing the V3–V4 region of the 16S rDNA, but not always. Each hypervariable region is more specific for certain taxa, thus studies based on different hypervariable regions are difficult to compare. The advent of long reads-sequencing could overcome such difficulties. Long-reads sequencing will most likely also improve results generated by shotgun sequencing, improving assembly and prediction of gene function on taxonomic analysis. Shotgun metagenomics should be preferred for future investigations to gather comprehensive knowledge on GIM and *ARG*s with their genetic elements considering that in current longitudinal studies analyzing antibiotics effect on GIM, the *ARG*s analysis has been largely neglected.

This review, encompassing the main animal hosts and all antibiotic classes, provides inspiration for future investigations, highlighting the major knowledge gaps that need to be filled for improving antibiotic usage and mitigating negative effects of these drugs. Decorticating GIM composition and function will unveil revolutionary strategies for medication and improvement of animals’ health status, resulting in positive consequences on global health.

## Figures and Tables

**Table 1:** Summary of studies investigating impact of antibiotics on bovines’ intestinal microbiota and relative antibiotic resistance genes.

Antibiotic	Farm	Administration	Dose (mg/kg of Animal)	Duration (Days)	Method	Effect on GIM	Effect on *ARG*s	Reference
Thiamphenicol	Exp	SCI	40	1	16S rDNA (V3–V4), shotgun, qPCR	Proteobacteria, Archaea *Prevotellaceae* ↑	*mcr*-2, *oqx*B ↑	[6]
Neomycin	Exp	O	10 mg/day	50	qPCR	ND		[13]
Oxytetracycline			1000 mg/day	14			*tet*O ↑	
Cefalexin	Comm	O	ND	14	16S rDNA (V3–V4)	Chlamydiae ↑	ND	[46]
Cefquinome	Exp	O	2 mg/L	3	Cultivation	ESBL*-*producing *E.coli* ↑	*bla*_CTX-M-1_ ↑	[47]
			20 mg/L					
Beta-lactams	Exp	O	ND	42	16S rDNA (V1–V3)	None	ND	[48]
Ceftiofur	Exp	O	0.1 mg/L	42	16S rDNA (V4)	*Veillonella ↓*	ND	[50]
Penicillin			0.005 mg/L					
Ampicillin			0.01 mg/L					
Oxytetracycline			0.3 mg/L					
Benzyl-penicillin	Exp	IMI	40,000 IU	6–14	Cultivation, ARISA and TRFLP	None	PEN resistance ↑	[51]
Trimethoprim-sulfamethazole	Exp	O	0.7 mL/10 kg	3	16S rDNA (V1–V3)	Diversity ↓ Intra-individual variability *↑*	ND	[52]
Amoxicillin	Comm	IMI	15	5–26	qPCR	Abundance ↓	*bla*_TEM_, *str*A/B, *tet*A*, int*I1 ↑	Personal data
Ceftiofur	Exp	SCI	0.2 mL/10 kg	1	qPCR		ND	[55]
Tulathromycin			2.5			*Bifidobacterium* *↓*		
Tulathromycin	Comm	SCI	7.5 and 12.5	1	Shotgun		*erm*A *↑*	[57]
Enrofloxacin			2.5			Desulfovibrionales *↑*	*gyr*A mutation ↑	
Tildipirosin	Exp	SCI	4	1	16S rDNA (V4), qPCR	None	ND	[58]
Multiple	Comm	O	ND	1–10	16S rDNA (V4), qPCR	Diversity, *E. coli ↓*	ND	[59]
Oxytetracycline	Exp	O	2 g/day	5	16S rDNA (V4) and shotgun	*Ruminococcus*, *Coprobacillus*, *Lachnospiraceae* ↓ *Prevotella*, *Faecalibacterium*, *Blautia* ↑	*tetM*, *mel* and *floR* ↑	[60]
			0.1–0.2 mg/day	42			None	
Oxytetracycline	Exp	IMI	20	ND	16S rDNA (V1–V2)	*Lactobacillus* ↓	ND	[61]
Tulathromycin		SCI	40			None		
Florfenicol								
Marbofloxacin	Comm	IMI	2 and 10	1	Cultivation	None	FQ resistant Enterobacterales ↑	[62]
Oxytetracycline	Exp	IMI	20	1	16S rDNA (V4), qPCR	Diversity, abundance *↓*	*tet*M, *tet*W ↑	[64] *
Tulathromycin			2.5	5–14		*Dialister, Oscillospira, Roseburia*, *Lachnospiraceae* ↓		

Note. GIM: gastro-intestinal microbiota; *ARG*s: antibiotic resistance genes; Exp: experimental; O: oral; ND: not determined; IMI: intramuscular injection; PEN: penicillin; Comm: commercial; ESBL: extended spectrum beta-lactamase; SCI: sub-cutaneous injection; FQ: fluoroquinolones. * this study was conducted on adult animals, whereas all the others were conducted on calves. ↑: increase; *↓*: decrease.

**Table 2:** Summary of studies investigating impact of antibiotics on pigs’ intestinal microbiota and relative antibiotic resistance genes.

Antibiotic	Farm	Administration	Dose (mg/kg of Animal)	Duration (Days)	Method	Effect on IM	Effect on *ARG*s	Reference
Oxytetracycline	Exp	O	5	7	16S rDNA (V4), and qPCR	Fibrobacteres, Proteobacteria ↓ Euryarchaeota, Actinobacteria ↑	*tetW*, *aph2′-id* ↑	[16]
		IMI	4					
Amoxicillin	Exp	IMI	15	1	DGGE of 16S rDNA	Diversity, abundance *↓*	ND	[71]
Tilmicosin	Exp	O	400	ND	DGGE of 16S rDNA	Abundance, Enterobacterales ↓ Lactobacilli ↑	ND	[72]
Amoxicillin			600			Lactobacilli ↓ Enterobacterales ↑		
Doxycycline			300					
Amoxicillin	Exp	O	30	14	Cultivation, 16S rDNA	Diversity, Firmicutes ↓ Proteobacteria↑	ND	[73]
Amoxicillin	Exp	PAR	15	5	Cultivation, 16S rDNA (V3–V4)	*Lactobacillus* ↓	ND	[74]
		O	12–20					
Ampicillin	Exp	O	20	7	qPCR	ND	*bla*_TEM_ ↑	[75]
		IMI						
Amoxicillin	Exp	PAR	20	7	Shotgun	*Lactobacillus*, *Faecalibacterium*, *Megasphaera*, *Oxalobacter* ↓ Enterobacterales, *Bacteroides*, *Fusobacterium* ↑	*cfx*A, *bla*_TEM,_ *aph*4-1a, *sat-2a*, *sph*, *str*A/B, *mph*E, *sul*2, *tet*B, *tet*Y ↑	[76]
Ertapenem		IVI	50			*Faecalibacterium*, *Megasphaera*, *Oxalobacter* ↓ *Bacteroidetes*, *Pseudomonas, Enterococcus*, *Acinetobacter* ↑	*bla*_IMP-27_, *aph*4-1a, *SAT*-2a*, sph, str*A/B, *mph*E, *sul*2, *tet*B, *tet*Y ↑ *dfr*A5/12 ↓	
Amoxicillin	Exp	IMI or O	ND	5	Cultivation	ND	AMX*-*resistant *E. coli* ↑	[77]
Ceftiofur	Exp	PAR	0.5–5	3	Cultivation	ND	Resistant *E. coli* ↑	[78]
		O	1–10	3				
Enrofloxacin		PAR	0.5–5	7				
Amoxicillin	Comm	IMI	150 mg/ml	1	Cultivation	ND	None	[79]
Tulathromycin	Exp	IMI	2.5	1	Shotgun sequencing	None	None	[80]
Lincomycin	Comm	O	1.000	7–14	16S rDNA (V3–V4)	Diversity, abundance, Spirochetes, Bacteroidetes ↓ Firmicutes, Actinobacteria ↑	ND	[81]
Oxytetracycline	Exp	O	40	14	Metagenomic shotgun sequencing	Diversity, richness, Firmicutes ↓ Bacteroidetes, Proteobacteria ↑	Enrichment, diversity ↑	[82]
Chlortetracycline	Exp	O	75	90	16S rDNA (V3–V4), and qPCR	*Lactobacillus*, *Pseudoalteromonas* ↑, *Prevotella*, *Sphaerochaeta*, *Shuttleworthia* ↓	*tetC*, *tetG*, *tetW* and *sul1*↑	[83]
Chlortetracycline	Exp	O	400	12	16S rDNA (V4)	Diversity, richness, *Lactobacillus*, *Succinivibrio* ↓	ND	[84]
Chlortetracycline	Exp	O	100	10	16S rDNA (V3–V4)	Verrucomicrobia ↓	ND	[85]
Ceftiofur (FA)	Exp	IMI	5	ND	16S rDNA (V1–V3)	Firmicutes/Bacteroidetes ratio: ↑	ND	[86]
Ceftiofur (Na)			5			↑		
Oxytetracycline			4			↓		
Penicillin			15.000 UI/lb			↓		
Tulathromycin			2.5			↑		
Colistin	Exp	O	50.000 UI/kg	5	16S rDNA (V4)	*E*. *coli*, *Shigella* ↓	ND	[87]
Cocktail	Exp/Comm	O	50.000 UI/kg	5	16S rDNA (V4), qPCR, cultivation	Enterobacterales, *Enterococcaceae* ↑	ND	[88]
			3.600 UI/kg					
Cocktail	Comm	ND	Multiple doses	1–66	16S rDNA (V1–V3), cultivation	Firmicutes ↑ Bacteroidetes ↓	*E.coli* and *Enterococcus* MDR ↑	[90]
Flavomycin	Comm	O	5	56	16S rDNA (V3–V4)	Proteobacteria, Fibrobacteres ↓	ND	[91]
Enramycin			15					
Tylosin	Exp	O	100	39	16S rDNA (V3–V4)	Firmicutes/Bacteroidetes ↑ Tenericutes ↓	ND	[92]

Note. GIM: gastro-intestinal microbiota; *ARG*s: antibiotic resistance genes; Exp: experimental; O: oral; ND: not determined; IMI: intra-muscular injection; DGGE: denaturing gradient gel electrophoresis; PAR: parenteral; Comm: commercial; IVI: intravenous injection; MDR: multidrug resistant; ↑: increase; *↓*: decrease.

**Table 3:** Summary of studies investigating impact of antibiotics on chickens’ intestinal microbiota and relative antibiotic resistance genes.

Antibiotic	Farm	Administration	Dose (mg/kg of Animal)	Duration (Days)	Method	Effect on GIM	Effect on *ARGs*	Reference
Tetracycline	Exp	O	60	7	16S rDNA (V3–V4), qPCR	Bifidobacteriales, Bacteroidales, Clostridiales, Desulfovibrionales, Burkholderiales, Campylobacterales ↓ Enterobacterales, Lactobacillales ↑	ND	[93]
Streptomycin			15	2				
Bacitracin	Exp	O	50	35	16S rDNA (V1–V3)	Diversity ↓ (caecum)	ND	[95] *
			200	77				
Penicillin	Exp	O	55	18	Pyrosequencing, qPCR	Firmicutes ↑ Bacteroidetes ↓	ND	[96]
Ampicillin	Exp	IMI or O	300	5	qPCR, 16S rDNA, (V4–V5), shotgun	Proteobacteria ↑	None	[97]
Virginiamycin	Exp	O	30	28	16S rDNA (V3–V4)	Diversity, richness, Firmicutes ↓ Bacteroidetes ↑	ND	[98]
Virginiamycin	Exp	O	20	50	Cultivation	*Lactobacillus*, *Clostridioites, Globicatella*, *Enterococcus*, *Corynebacterium* ↑	ND	[99]
Monesin	Comm	O	110	14	16S rDNA (V3), shotgun	Firmicutes ↓ Proteobacteria ↑	None	[100]
Virginiamycin			110					
Tylosin			15–20					
Chlortetracycline	Comm	O	100	42	16S rDNA (V1–V9)	*Lactobacillus*, *Megamonas*, *Helicobacter* ↑ *Alistipes* ↓	ND	[101]
Amoxicillin	Exp	O	0.50 µg/kg	42	Cultivation, 16S rDNA (V1–V2)	Firmicutes/Bacteroidetes ↑	ND	[102]
Chlortetracycline			0.1					
Virginiamycin			0.015					
Enrofloxacin	Exp	O	10 or 100	7	16S rDNA (V3–V4)	*Anaerotruncus, Butyricicoccus, Ruminococcus* ↓	ND	[103]
Enrofloxacin	Exp	O	10	7	16S rDNA (V4)	*Anaerotruncus, *Blautia*, *Janibacter*, *Flavisolibacter*, *Parasutterella** ↓	ND	[104]
			100			Proteobacteria,**Bacillus*, *Lactococcus** ↑ *Anaerotruncus, *Blautia*, *Janibacter*, *Flavisolibacter*, *Parasutterella** ↓		
Amoxicillin	Exp	O	5	5	16S rDNA (V1–V9)	Diversity ↓	ND	[105]
Enrofloxacin			11					
Enrofloxacin/Diclazuril	Exp	O	10/0.3	14	16S rDNA (V4)	Firmicutes, Actinobacteria, Thermi, Verrucomicrobia ↓	ND	[106]
Bacitracin	Exp	O	1000	30	16S rDNA (V3–V4)	Richness, Firmicutes ↓ Bacteroidetes ↑	ND	[107]
Bacitracin	Exp	O	200	7	16S rDNA (V3–V5)	Caecum: Clostridia ↑, *Peptostreptococcaceae* ↓ Distal colon: *Oscillospira*, *Erysipelotrichaceae* ↓, *Lachnospiraceae* ↑	ND	[108]
Avilamycin	Exp	O	25	35	16S rDNA (V1–V3)	Diversity ↓ (ileum)	ND	[109]
Amoxicillin	Exp	O	1.430	22	16S rDNA (V3–V4)	*Enterococcaceae* ↑	ND	[110]
Thiamphenicol			0.2					

Note. GIM: gastro-intestinal microbiota; *ARGs*: antibiotic resistance genes; Exp: experimental; O: oral; Nd: not determined; IMI: intra-muscular injection; *: this study was conducted on turkeys; Comm: commercial; ↑: increase; *↓*: decrease.

**Table 4:** Summary of studies investigating impact of antibiotics on companion animals’ intestinal microbiota and relative antibiotic resistance genes.

Host	Antibiotic	Farm	Administration	Dose (mg/kg of Animal)	Duration (Days)	Method	Effect on GIM	Effect on *ARGs*	Reference
	Penicillin	ND	IMI	20.000 UI/kg	5	16S rDNA (V4)	Diversity, richness, Verrucomicrobia ↓	ND	[7]
	Ceftiofur		O	2.2					
	Trimethoprim/sulfadiazine			30					
Horses	Benzyl-Penicillin	ND	IMI	20.000 UI/kg	5	Cultivation, DGGE-16S rDNA (V3)	Bacteroidetes, *Clostridioites*, *Enterococcaceae* ↑	None	[113]
	Trimethoprim/sulfadiazine	ND	O	30	7	Cultivation	*Lactobacillaceae*, cellulolytic bacteria ↓ *Salmonella*, *C. difficile* ↑	ND	[114]
	Ceftiofur		IMI	2.2					
	Erythromycin Rifampicin	ND	O	30	14	16S rDNA (V4), shotgun, cultivation	Diversity, abundance, *Rhodococcus equi* ↓	*ARG*s macrolides, rifampin, doxycycline ↑	[115]
	Metronidazole	ND	O	30	3	16S rDNA (V4)	Diversity ↓	ND	[116]
	Multiple	ND	ND	Multiple	2–14	16S rDNA (V4)	Fusobacteria↓ Tenericutes ↓ WPS-2 * ↓	ND	[117]
Dogs	Metronidazole	ND	O	30	14	16S rDNA (V4), qPCR	Diversity, richness, Bacteroidetes, Fusobacteria, Clostridiales ↓ Proteobacteria, Actinobacteria, Lactobacillales ↑	ND	[119]
	Metronidazole, prednisolone	Exp	O	25/1	14	16S rDNA (V4)	Diversity, richness, Fusobacteria Clostridiales ↓ Actinobacteria, Bacilli ↑	ND	[120]
	Metronidazole/spiramycin	ND	O	12.5/7.500 UI/kg	6	qPCR	None	ND	[121]
	Tylosin	ND	O	40	14	16S rDNA (V4), qPCR	*Enterococcaceae* ↑ *Fusobacteriaceae*, *Veillonellaceae*, *Bacteroidaceae* ↓	ND	[122]
	Tylosin	Exp	ND	20–22	14	16S rDNA (V4–V5)	Diversity, richness, Fusobacteria, Bacteroidales, *Moraxella* ↓ Enterococci, *Pasteurella* spp*., Dietzia* spp.↑	ND	[123]
	Amoxicillin	Exp	O	20	7	DGGE-16S rDNA (V3), qPCR	Enterobacterales ↑	AMX-resistant *E. coli* ↑	[124]
	Amoxicillin/clavulanic acid	ND	O	25–50	7	Cultivation, qPCR	None	AMX-resistant *E. coli* ↑	[125]
	Amoxicillin	ND	O	10–20	5–13	Cultivation, 16S rDNA (V3–V4)	Diversity, richness, Firmicutes ↓ Proteobacteria ↑	AMX-resistant Enterococci and *E. coli* ↑	[126]
	Amoxicillin/clavulanic acid				5–14		*Dialister, Oscillospira, Roseburia*, *Lachnospiraceae* ↓		
Cats	Clindamycin	Exp	O	12.1–22.7	21	16S rDNA (V4), qPCR	*Actinobacteria,Bacteroidetes,Ruminococcaceae,Veillonellaceae,Erysipelotrichaceae↓Clostridiaceae,Proteobacteria* ↑	ND	[127]

Note. GIM: gastro-intestinal microbiota; *ARGs*: antibiotic resistance genes; ND: not determined; IMI: intra-muscular injection; DGGE: Denaturing Gradient Gel Electrophoresis; O: oral; *: candidate phylum (Eremiobacterota); Exp: experimental; AMX: amoxicillin.

## Data Availability

Data are available to be shared at any request.
